# Alphavirus Evasion of Zinc Finger Antiviral Protein (ZAP) Correlates with CpG Suppression in a Specific Viral nsP2 Gene Sequence

**DOI:** 10.3390/v15040830

**Published:** 2023-03-24

**Authors:** LeAnn P. Nguyen, Kelly S. Aldana, Emily Yang, Zhenlan Yao, Melody M. H. Li

**Affiliations:** 1 Molecular Biology Institute, University of California, Los Angeles, Los Angeles, CA 90095, USA; 2 Department of Microbiology, Immunology and Molecular Genetics, University of California, Los Angeles, Los Angeles, CA 90095, USA; 3 AIDS Institute, David Geffen School of Medicine, University of California, Los Angeles, Los Angeles, CA 90095, USA

**Keywords:** alphavirus, ZAP, RNA binding, CpG sensing

## Abstract

Certain re-emerging alphaviruses, such as chikungunya virus (CHIKV), cause serious disease and widespread epidemics. To develop virus-specific therapies, it is critical to understand the determinants of alphavirus pathogenesis and virulence. One major determinant is viral evasion of the host interferon response, which upregulates antiviral effectors, including zinc finger antiviral protein (ZAP). Here, we demonstrated that Old World alphaviruses show differential sensitivity to endogenous ZAP in 293T cells: Ross River virus (RRV) and Sindbis virus (SINV) are more sensitive to ZAP than o’nyong’nyong virus (ONNV) and CHIKV. We hypothesized that the more ZAP-resistant alphaviruses evade ZAP binding to their RNA. However, we did not find a correlation between ZAP sensitivity and binding to alphavirus genomic RNA. Using a chimeric virus, we found the ZAP sensitivity determinant lies mainly within the alphavirus non-structural protein (nsP) gene region. Surprisingly, we also did not find a correlation between alphavirus ZAP sensitivity and binding to nsP RNA, suggesting ZAP targeting of specific regions in the nsP RNA. Since ZAP can preferentially bind CpG dinucleotides in viral RNA, we identified three 500-bp sequences in the nsP region where CpG content correlates with ZAP sensitivity. Interestingly, ZAP binding to one of these sequences in the nsP2 gene correlated to sensitivity, and we confirmed that this binding is CpG-dependent. Our results demonstrate a potential strategy of alphavirus virulence by localized CpG suppression to evade ZAP recognition.

## 1. Introduction

Alphaviruses are positive-sense, single-stranded RNA viruses of the *Togaviridae* family that are transmitted by arthropod vectors [1]. Alphaviruses are classified into two lineages, Old World and New World, with different disease manifestations [2]. Diseases caused by Old World alphaviruses range in severity: the Ross River virus (RRV) and the Sindbis virus (SINV) typically cause less serious symptoms such as rash and fever, while infection with the more pathogenic chikungunya virus (CHIKV) can result in acute joint pain and persistent arthritis that can last from months to years [2]. CHIKV has re-emerged on multiple continents and caused widespread outbreaks in the 21st century [3]. During these outbreaks, a high proportion of CHIKV-infected individuals also displayed neurological disease [4]. The geographic ranges of alphaviruses continue to expand due to global warming, urbanization, and intercontinental travel [5,6], but no virus-specific therapies currently exist to treat or prevent alphavirus infection [7]. To develop such therapies, a greater understanding of the mechanisms underlying alphavirus pathogenesis and virulence is needed.

One viral strategy of increased virulence is the evasion of immune defenses, such as the type I (α/β) interferon (IFN) response. Production of IFN upon the cellular detection of viral invasion stimulates the expression of antiviral genes known as IFN-stimulated genes (ISGs). One important ISG product is zinc finger antiviral protein (ZAP), an RNA-binding protein that inhibits a broad range of RNA and DNA viruses [8]. Specifically, ZAP inhibits alphaviruses by blocking viral translation in concert with its co-factor TRIM25 [9,10]. ZAP can also destabilize viral RNA to inhibit viruses such as human immunodeficiency virus 1 (HIV-1), Japanese encephalitis virus (JEV), Ebola virus, hepatitis B virus, human cytomegalovirus, and coxsackievirus B3 [11,12,13,14,15,16], which requires the recruitment of various other cellular co-factors [17,18,19]. However, many viruses are resistant to inhibition by ZAP, including vesicular stomatitis virus, poliovirus, yellow fever virus (YFV), dengue virus (DENV), Zika virus (ZIKV), and herpes simplex virus type 1 (HSV-1) [11,20]. Particularly intriguing is the fact that ZAP can inhibit certain viruses within a family but not others, such as for the *Flaviviridae* family, where ZAP can inhibit JEV but not YFV, DENV, or ZIKV [11,20]. This suggests that the determinants of ZAP inhibition are very virus-specific and not necessarily shared, even among closely related viruses. Some ZAP-resistant viruses are known to encode proteins that actively antagonize ZAP’s antiviral activity [21,22,23], but in other cases, it is unclear if ZAP-resistant viruses evade ZAP inhibition by viral-encoded antagonism activity or masking of ZAP recognition motifs in viral RNA. These latter cases highlight how the exact viral determinants that sensitize a virus to ZAP inhibition remain poorly understood.

ZAP recognition and binding of viral RNA is essential for its antiviral activity [24]. ZAP is expressed as at least four splice isoforms [25], all of which share an N-terminus with four CCCH zinc fingers that directly bind viral RNA [8,26]. The two most well-characterized isoforms are short (ZAPS) and long (ZAPL), with ZAPS expression being more IFN-inducible while ZAPL is expressed constitutively [14,27,28]. The common N-terminus of all ZAP isoforms is sufficient for its antiviral activity due to the RNA binding ability of the CCCH zinc fingers [11,24], but the C-terminal catalytically-inactive poly(ADP-ribose) polymerase (PARP)-like domain of ZAPL confers additional antiviral potency [28,29,30]. Studies of ZAP in the context of HIV-1 suggest that it preferentially binds CpG dinucleotides in HIV-1 RNA [31], a preference further supported by structural studies of ZAP bound to CpG-containing RNA [32,33]. Mutation of ZAP residues important for CpG recognition also has a detrimental effect on its ability to inhibit SINV [34]. Interestingly, one study found that while the overall CpG dinucleotide contents of primate lentivirus genomes do not correlate with their ZAP sensitivities, there is a correlation between CpG content within a specific ~700-bp window of the HIV-1 *env* gene and the ZAP sensitivity of HIV-1 strains [35]. This suggests that ZAP binding to CpG dinucleotides may be dependent on their context within viral RNA. In support of this, another study found that the ZAP sensitivity of HIV-1 mutants is influenced by the number, spacing, and flanking nucleotide context of CpG dinucleotides in the *env* gene [36]. Other studies indicate that ZAP may also recognize cytosine-rich sequences [15] and UpA dinucleotides [37]. Further characterization of the mechanisms of ZAP viral RNA recognition would provide insight into the evolutionary pressures that ZAP exerts on viruses.

Recently, we observed that Old World alphaviruses exhibit differential sensitivity to inhibition by individual ZAP isoforms: RRV and SINV are more sensitive to ZAP than CHIKV and its close relative, o’nyong’nyong virus (ONNV) [25]. In this study, we sought to use this panel of related alphaviruses with a range of ZAP sensitivities to probe viral determinants of ZAP susceptibility. Since our previous experiments were conducted in ZAP-deficient cell lines with induced expression of individual isoforms, which might take on different antiviral activities when co-expressed, we further characterized alphavirus sensitivity to endogenous ZAP isoforms at baseline and upon IFN stimulation. We found a similar pattern of differential alphavirus sensitivity to endogenous ZAP, apart from CHIKV production being inhibited by endogenous ZAP but not individual ZAP isoforms. We also assayed in vitro ZAP binding to alphavirus genomic RNA and did not find a correlation with ZAP sensitivity. Using a chimeric virus, we determined that the non-structural protein (nsP) gene region of the alphavirus genome largely contains the ZAP sensitivity determinant, but again we did not find a correlation between ZAP binding to this region and alphavirus sensitivity. Because we previously did not find any obvious relationships between ZAP sensitivity and genomic CpG content of alphaviruses [25], here we analyzed CpG content in 500-bp sliding windows along each alphavirus genome. We identified three windows in the nsP region where CpG content correlates with ZAP sensitivity. Interestingly, binding to only one window in the nsP2 gene correlates to sensitivity, and mutagenesis of this window confirmed that this binding is CpG-dependent. Our results indicate that ZAP only recognizes CpG dinucleotides within specific contexts of alphavirus RNA, consistent with findings for HIV-1. ZAP-resistant alphaviruses may thus evade ZAP inhibition by suppressing CpG content in localized regions important for recognition. Our work further illuminates the mechanism by which ZAP recognizes alphaviruses, as well as one potential strategy of alphavirus escape from immune restriction.

## 2. Materials and Methods

### 2.1. Cells

Zinc finger nuclease-mediated ZAP knockout (KO) 293T cells (clone 89) and the parental wild-type (WT) 293T cells were generously provided by Dr. Akinori Takaoka at Hokkaido University, Sapporo, Japan [27]. ZAP KO 293T cells with inducible expression of red fluorescent protein (RFP)-tagged ZAPS or ZAPL were generated as previously described, and ZAP expression was induced by treatment with 1 μg/mL doxycycline for 24 h [25]. WT 293T cells, ZAP KO 293T cells, and ZAP KO 293T cells with inducible expression of RFP-tagged ZAPS or ZAPL were cultured in Dulbecco’s modified Eagle’s medium (DMEM) supplemented with 10% fetal bovine serum (FBS). Baby hamster kidney 21 (BHK-21, ATCC) cells were cultured in minimum essential medium (MEM) supplemented with 7.5% FBS. Where indicated in the text, cells were treated with 5 U/mL or 10 U/mL IFN-β (PeproTech, Cranbury, NJ, USA) in the culture medium.

### 2.2. Plasmids, Viruses, and Infections

SINV-expressing enhanced green fluorescent protein (EGFP) (TE/5′2J/GFP), ONNV SG650 strain expressing EGFP (generously provided by Dr. Steve Higgs, Kansas State University, Manhattan, KS, USA) (ONNV-GFP), RRV T48 strain (generously provided by Dr. Richard Kuhn, Purdue University, West Lafayette, IN, USA), RRV expressing EGFP (generously provided by Dr. Mark Heise, the University of North Carolina, Chapel Hill, NC, USA), and CHIKV vaccine strain 181/clone 25 (generously provided by Dr. Scott Weaver, the University of Texas Medical Branch, Galveston, TX, USA) have been previously described [38,39,40,41,42]. CHIKV with expression of EGFP under the control of the subgenomic promoter was generated by three fragment ligations using the NEBuilder HiFi DNA Assembly kit (New England Biolabs, Ipswich, MA, USA), according to the manufacturer’s instructions. CHIKV vaccine strain 181/clone 25 was digested with SwaI and XmaI, and ligated with the pre-GFP, EGFP, and post-GFP fragments. The pre- and post-GFP fragments were amplified from CHIKV strain 181/clone 25 with primers to add overlapping regions with GFP (Table S1). The EGFP fragment was amplified from ONNV-GFP with primers to add overlapping regions with the pre- and post-GFP fragments (Table S1).

The chimeric SINV/ONNV viruses were derived from TE/5′2J/GFP and ONNV-GFP. The *cis*-acting RNA elements and nonstructural protein (nsP) genes in the viral plasmid backbones were obtained from SINV by digestion with XbaI (5′) and XhoI (3′) and from ONNV by digestion with AscI (5′) and NotI (3′), followed by gel extraction with the Zymoclean Gel DNA Recovery Kit (Zymo Research, Irvine, CA, USA). The subgenomic promoter, EGFP, structural protein (sP) genes, 3′ untranslated region, and poly (A) tail were amplified from SINV with primers to add 5′ AscI (5′-GTATGCGCGCGCCACCATGGTGAGCAAG-3′) and 3′ NotI (5′-gtttGCGGCCGCATTCCCCTCGAGGAATTCCC-3′) sites and from ONNV with primers to add 5′ NheI (5′-GTTTGCTAGCGCCACCATGGTGAGCAAGGGCG-3′, compatible with XbaI ligation sites) and 3′ XhoI (5′-GTTTCTCGAGCCTCGATTAATTAAGCGGCCGC-3′) sites. Primers were synthesized by Integrated DNA Technologies and PCR was performed with Q5 High-Fidelity DNA Polymerase (New England Biolabs). The resulting PCR products were digested and cloned into the corresponding viral plasmid backbones using T4 DNA ligase (New England Biolabs).

Virus stocks were generated in BHK-21 cells as previously described [20]. Titers for multiplicity-of-infection (MOI) calculations and virion production by 293T cell lines were assayed in BHK-21 cells as previously described [20]. Viral infections were performed as previously described [20] and GFP-positive infected cells were analyzed on a Miltenyi Biotec MACSQuant Analyzer 10 Flow Cytometer.

### 2.3. In Vitro Transcription and Biotinylation

Transcription of biotinylated genomic SINV RNA and firefly luciferase (Fluc) RNA was performed as previously described using Sp6 RNA polymerase (New England Biolabs) and the mMESSAGE mMACHINE T7 Transcription Kit (Invitrogen, Waltham, MA, USA) [34], respectively. DNA templates for genomic RNA transcription were generated for RRV by SacI linearization [42] and for ONNV and CHIKV by NotI linearization, followed by transcription of biotinylated RNA with Sp6 RNA polymerase as previously described [34,39,40,43]. To generate DNA templates for Sp6 RNA polymerase transcription of biotinylated nsP RNA using the same method as for genomic RNA, the *cis*-acting RNA elements and nsPs of SINV and ONNV were generated by restriction enzyme digestion as described in Section 2.2 above. *Cis*-acting RNA elements and nsPs were amplified from RRV using the primers 5′-TCGCCACCTCTGACTTGAGC-3′ and 5′-GTTTACTGTTGTGAGCTGTATTAGATGAAGG-3′ and from CHIKV using the primers 5′-GCTCGATTTAGGTGACACTATAG-3′ and 5′-TATGGCTGATTGGTATTTAGGTAC-3. DNA templates for the transcription of biotinylated CpG-correlated window RNA were amplified using primers to generate templates of length 500 ± 2-bp (to facilitate amplification and transcription without altering CpG content) with a 5′ T7 promoter sequence (Table S2). The windows were transcribed in vitro using the HiScribe T7 Quick High Yield RNA Synthesis Kit (New England Biolabs) supplemented with 2.5 mM biotin-16-UTP (Roche Life Science, Penzberg, Germany). RNA biotinylation was confirmed by streptavidin-HRP dot blot [44] (Figure S1). Briefly, 50 ng of biotinylated RNA diluted in RNAse-free water was dotted onto a positively charged nylon membrane (Thermo Fisher Scientific, Waltham, MA, USA) primed with 10× SSC buffer (1.5 M NaCl, 150 mM NaCit, HCl to pH 7.0), then crosslinked using 254 nm ultraviolet light. The membrane was incubated for 30 min in blocking buffer (120 mM NaCl, 16 mM Na_2_HPO_4_, 8 mM NaH_2_PO_4_, 170 mM SDS), incubated for 1 h with streptavidin-HRP antibody (catalog number N100; Thermo Fisher Scientific) diluted 1:300 in blocking buffer, and then washed twice with wash buffer A (1:10 blocking buffer) for 30 min and twice with wash buffer B (100 mM Tris pH 9.5, 100 mM NaCl, 20 mM MgCl_2_) for 5 min. Biotinylated RNA was visualized with ProSignal Pico ECL Reagent (Genesee Scientific, El Cajon, CA, USA) on a ChemiDoc imager (Bio-Rad, Hercules, CA, USA).

### 2.4. In Vitro RNA Pull-Down Assay

In vitro RNA pull-down was performed as previously described [34]. 0.4 pmol of biotinylated RNA was used for the genomic and nsP RNA pull-down experiments, which was determined to be the minimal amount of RNA required to see ZAP binding to SINV RNA. An amount of 1 pmol of biotinylated RNA was used for the CpG-correlated window RNA pull-down experiments to account for the shorter length of the windows relative to genomic and nsP RNA. Immunoblots for the genomic and nsP RNA experiments were quantified by ImageJ as previously described [45].

### 2.5. Immunoblotting

Total protein was isolated by cell lysis using RIPA lysis buffer (150 mM NaCl, 1% NP-40, 0.5% sodium deoxycholate, 0.1% SDS, and 50 mM Tris-HCl) supplemented with cOmplete Mini Protease Inhibitor Cocktail (Roche Life Science), followed by quantification with a colorimetric protein assay (Bio-Rad). Proteins were resolved by SDS-PAGE using Tris-glycine-SDS electrophoresis buffer (25 mM Tris, 192 mM glycine, and 0.1% SDS) and 4–15% precast Mini-PROTEAN TGX Gels (Bio-Rad) before transferring to nitrocellulose membranes (Bio-Rad). Immunodetection was performed with 1:5000 anti-ZAP (catalog number ab154680; Abcam, Cambridge, UK) and 1:20,000 anti-actin-HRP (catalog number A3854; Sigma-Aldrich, St. Louis, MO, USA). The ZAP primary antibody was detected with 1:20,000 goat anti-rabbit-HRP (Thermo Fisher Scientific). Proteins were visualized with ProSignal Pico ECL Reagent (Genesee Scientific) on a ChemiDoc imager (Bio-Rad).

### 2.6. Quantitative Reverse Transcription PCR (RT-qPCR)

Total RNA was isolated by Quick-RNA kit (Zymo Research). An amount of 0.8 μg of input RNA was used as a template for reverse transcription by ProtoScript II First Strand cDNA Synthesis Kit (New England Biolabs) and random hexamers. RT-qPCR was performed with 5 μL of 8-fold diluted cDNA and primers targeting ISG15 or RPS11 (Table S3) using the Luna Universal qPCR Master Mix (New England Biolabs). qPCR was performed on the CFX Real-Time PCR system (Bio-Rad) courtesy of the UCLA Virology Core, with conditions as follows: initial denaturation at 95 °C for 1 min, then 40 cycles of 95 °C for 15 s and 60 °C for 30 s, and then a final 10 s at 60 °C. A melt curve was then calculated by heating from 60 °C to 95 °C with increases of 0.5 °C/s for 10 s at each temperature. mRNA levels of ISG15 were calculated by normalizing the target transcript Ct value to the Ct value of the housekeeping gene RPS11. ISG15 mRNA fold change was calculated with the RPS11-normalized values relative to the average values from ZAP KO 293T cells treated with 0 U/mL IFN-β for 0 h.

### 2.7. CpG Content Sliding Window Analysis and UpA Content Analysis

Sliding window analysis of alphavirus CpG content was performed using a custom Python script (https://github.com/nguyelea/alphavirus_zap_sensitivity_paper/blob/main/cg_sliding_window.py, accessed on 22 December 2022). In brief, the CpG observed/expected ratio was calculated by dividing the observed frequency of CpG dinucleotides within a window by the product of the frequencies of observed C and G mononucleotides within the same window. This calculation was performed on 500-bp sliding windows with a step size of 250-bp. The analysis was performed on the genomes of the wild-type alphavirus strains described in Section 2.2 above. Windows were aligned by Clustal Omega with default parameters [46].

UpA content analysis of windows with CpG contents correlating to ZAP sensitivity was performed using a custom Python script (https://github.com/nguyelea/alphavirus_zap_sensitivity_paper/blob/main/ua_obs_exp.py, accessed on 22 December 2022). The UpA observed/expected ratio was calculated by dividing the observed frequency of UpA dinucleotides by the product of the frequencies of observed U and A mononucleotides within each window.

### 2.8. Generation of CpG-Correlated Window 1 Mutants

Custom Python scripts were used to design mutants in which the CpG contents of RRV and SINV window 1 were depleted (CGlo mutants) (https://github.com/nguyelea/alphavirus_zap_sensitivity_paper/blob/main/cg_deplete.py, accessed on 22 December 2022) and the CpG contents of ONNV and CHIKV window 1 were enriched (CGhi mutants) (https://github.com/nguyelea/alphavirus_zap_sensitivity_paper/blob/main/cg_enrich.py, accessed on 22 December 2022). In brief, these scripts minimized or maximized the number of CpG dinucleotides in each sequence without altering overall GC content or maintaining amino acid coding sequences. Gene blocks of these mutants were synthesized by Integrated DNA Technologies and inserted into a Zero Blunt TOPO vector (Invitrogen) before amplification by PCR using primers to add a 5′ T7 promoter sequence (Table S2). Biotinylated RNA was transcribed as described in Section 2.2 for the CpG-correlated windows.

### 2.9. Statistical Analyses

All statistical analyses were performed using GraphPad Prism 9.

## 3. Results

### 3.1. Alphaviruses Show Differential Sensitivity to Endogenous ZAP in 293T Cells

**Figure 1:**
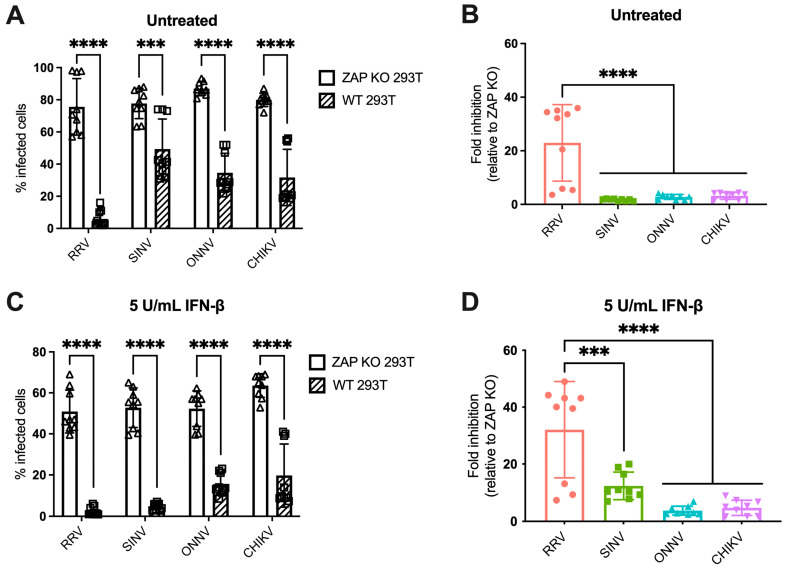
Alphavirus replication shows differential sensitivity to endogenous ZAP in 293T cells. (**A**,**C**) ZAP KO and WT 293T cells untreated (**A**) or treated with 5 U/mL IFN-β for 24 h (**C**) were infected with GFP-expressing RRV, SINV, ONNV, or CHIKV vaccine strain 181/clone 25 (MOI = 1 PFU/cell). Cells were harvested and fixed at 24 h post-infection (h.p.i.), and their percentage of infection was determined by flow cytometry. Data are combined from three independent experiments performed with biological replicates in triplicate wells. Error bars represent the standard deviations (SD). Asterisks indicate statistically significant differences (unpaired T test with Holm–Šídák’s multiple comparisons test: ***, *p* < 0.001; ****, *p* < 0.0001). (**B**,**D**) Fold inhibition of alphavirus replication by ZAP. Data from panels A (**B**) or C (**D**) are represented. Fold inhibition relative to ZAP KO was calculated by dividing the percentage of infection of each virus in ZAP KO 293T cells by the percentage of infection in WT 293T cells. Error bars represent the standard deviations (SD). Asterisks indicate statistically significant differences (one-way ANOVA and Tukey’s multiple comparisons test: ***, *p* < 0.001; ****, *p* < 0.0001).

We previously found differential ZAP inhibition of alphaviruses in ZAP KO 293T cells with doxycycline-inducible overexpression of each individual ZAP isoform tagged with red fluorescent protein (RFP) [25]. While this system was useful for studying the role of each ZAP isoform individually, it did not represent typical levels of ZAP expressed in 293T cells upon infection, nor could we observe the potential effect of interactions between multiple ZAP isoforms. We then asked if we would see a similar pattern of alphavirus sensitivity to all ZAP isoforms expressed together at endogenous levels in 293T cells. To do this, we infected ZAP KO 293T and the parental wild-type (WT) 293T cells with alphaviruses expressing green fluorescent protein (GFP) under the control of the subgenomic promoter, which provides a readout for genome replication. We then measured the percentage of GFP-positive (infected) cells by flow cytometry. The percentage of infected ZAP KO 293T cells is similar for all viruses when infected at a multiplicity of infection (MOI) of 1 plaque forming unit (PFU)/cell for 24 h, but while there is a decrease in infected cells in WT 293T cells compared to ZAP KO, the degree of decrease appears to be greater for RRV compared to the other viruses (Figure 1A). We indeed found that the fold inhibition of RRV replication in WT compared to ZAP KO 293T cells is significantly greater than that of the other alphaviruses, indicating that basal expression of ZAP has a greater inhibitory effect on RRV replication (Figure 1B). This is consistent with our previous finding that RRV is most sensitive to inhibition by overexpressed ZAP isoforms in our ZAP-inducible 293T cell lines [25].

IFN-β treatment upregulates all isoforms of ZAP in 293T cells, with the short and medium isoforms being most dramatically upregulated [25]. Thus, we asked whether pre-treating cells with IFN-β would boost endogenous ZAP expression to a degree that would further distinguish differences in alphavirus sensitivity to ZAP. We previously confirmed that treatment with IFN-β significantly upregulates ZAP protein expression in WT 293T cells, while there is no ZAP protein expression detected in ZAP KO 293T cells [25]. Here, we determined the optimal conditions of IFN-β treatment that would allow us to interrogate the effects of IFN-induced ZAP. We treated WT and ZAP KO 293T cells with 0, 5, and 10 units/mL (U/mL) of IFN-β for 6 and 24 h and measured percentage of cells infected with GFP-expressing SINV at each condition. We found that treatment with 5 U/mL of IFN-β for 24 h reveals the greatest range in the effect of ZAP inhibition when comparing SINV replication between ZAP KO and WT 293T cells, but still allows for a reasonable level of viral infection (Figure S2A). We also observed that following treatment with 5 U/mL IFN-β, mRNA expression of a canonical ISG, ISG15, is similar in WT and ZAP KO 293T cells (Figure S2B). This indicates that the difference in viral replication we see between ZAP KO and WT 293T cells is primarily due to ZAP expression in WT 293T cells rather than a more general difference in ISG expression between ZAP KO and WT 293T cells. Given our results, we decided to treat cells with 5 U/mL IFN-β for 24 h to assay the effect of IFN-induced ZAP on alphavirus replication.

Following 24 h of IFN-β treatment, we infected ZAP KO and WT 293T cells with GFP-expressing alphaviruses. The percentage of infected IFN-treated ZAP KO cells is similar across all viruses, although it is lower than that of infected untreated ZAP KO cells, demonstrating the antiviral effects of IFN-β (Figure 1A,C). There is a decrease in the percentage of infected IFN-treated WT compared to ZAP KO 293T cells for all viruses (Figure 1C). We found that the fold inhibition of RRV replication is again significantly greater than that of the other alphaviruses (Figure 1D). SINV also appears to be sensitive to inhibition by IFN-induced ZAP with an intermediate phenotype, while ONNV and CHIKV remain resistant to inhibition (Figure 1D). Interestingly, there appears to be a bimodal distribution of RRV biological replicates that we see both with and without IFN pre-treatment (Figure 1B,D). However, the data points that are low in both untreated and IFN-treated conditions are from the same independent experiment, suggesting that this distribution is a consequence of variability in infection efficiency across experiments. Taken together, our results suggest that while RRV replication is sensitive to both basal and IFN-induced endogenous ZAP, SINV replication is only sensitive to IFN-induced ZAP, and ONNV and CHIKV replication are resistant to both. The overall pattern of sensitivity is consistent with our previous findings in the ZAP-inducible 293T cell lines [25].

**Figure 2:**
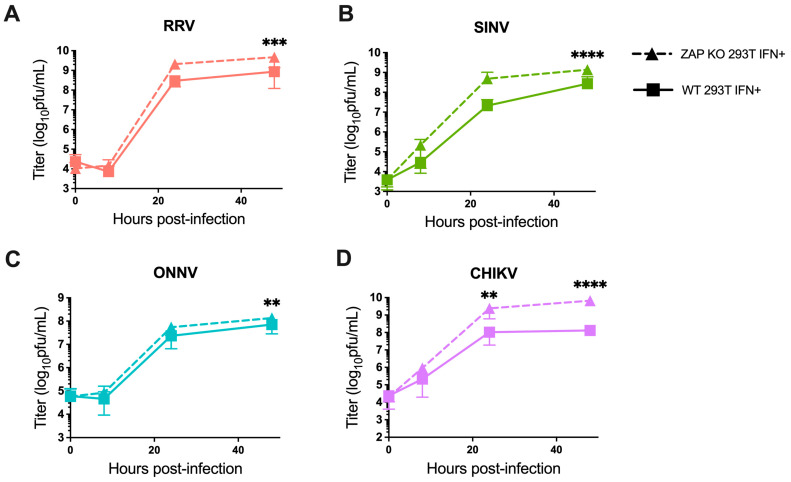
Alphavirus production shows differential sensitivity to IFN-induced endogenous ZAP in 293T cells. Following treatment with 5 U/mL IFN-β for 24 h, ZAP KO and WT 293T were infected with RRV (**A**), SINV (**B**), ONNV (**C**), and the CHIKV vaccine strain (181/clone 25) (**D**) at MOI = 0.1 PFU/cell. Media overlaying the cells were harvested at 0, 8, 24, and 48 h.p.i., and the viral titer was determined by infection of BHK-21 cells in standard plaque assays. Mean values from four biological replicates across two independent experiments are plotted, and error bars represent the SD. Asterisks indicate statistically significant differences (two-way ANOVA and Bonferroni posttest: **, *p* < 0.01; ***, *p* < 0.001; ****, *p* < 0.0001).

We also investigated the ability of endogenous ZAP in 293T cells to inhibit virion production of wild-type alphaviruses without GFP at different times post-infection. When we again pre-treated ZAP KO and WT 293T cells with 5 U/mL IFN-β for 24 h before infection, we found a significant decrease in RRV production at 48 h post-infection (h.p.i.) in WT compared to the ZAP KO 293T cells (Figure 2A). At 48 h.p.i., we also observed a significant decrease in SINV production in WT relative to ZAP KO 293T cells (Figure 2B). ONNV production is more similar in both WT and ZAP KO 293T cells except for a slight decrease at 48 h.p.i. (Figure 2C), but interestingly, CHIKV production shows a significant decrease at both 24 and 48 h.p.i. in WT compared to ZAP KO 293T cells (Figure 2D). This decrease in CHIKV production in the presence of ZAP in WT 293T cells is also apparent when we did not pre-treat cells with IFN-β, although we did not observe as much of a difference in production of the other viruses in non-IFN-treated WT versus ZAP KO 293T cells (Figure S3). These results suggest that endogenous ZAP can inhibit CHIKV at a viral life cycle step likely between genome replication and virion production.

Our findings indicate that the pattern of differential alphavirus sensitivity to ZAP is not only observed with overexpressed, RFP-tagged ZAP isoforms as we previously demonstrated but also with endogenous ZAP in 293T cells both with and without IFN pre-treatment. Generally, it appears that RRV and SINV replication and virion production are more sensitive to inhibition by ZAP while ONNV replication and production are relatively ZAP-resistant. Interestingly, CHIKV production but not replication is relatively sensitive to ZAP, suggesting a novel mechanism for ZAP inhibition of CHIKV that is not at the step of viral translation.

### 3.2. ZAPS and ZAPL Binding to Alphavirus Genomic RNA Do Not Correlate with Alphavirus Replication Sensitivity to ZAP

**Figure 3:**
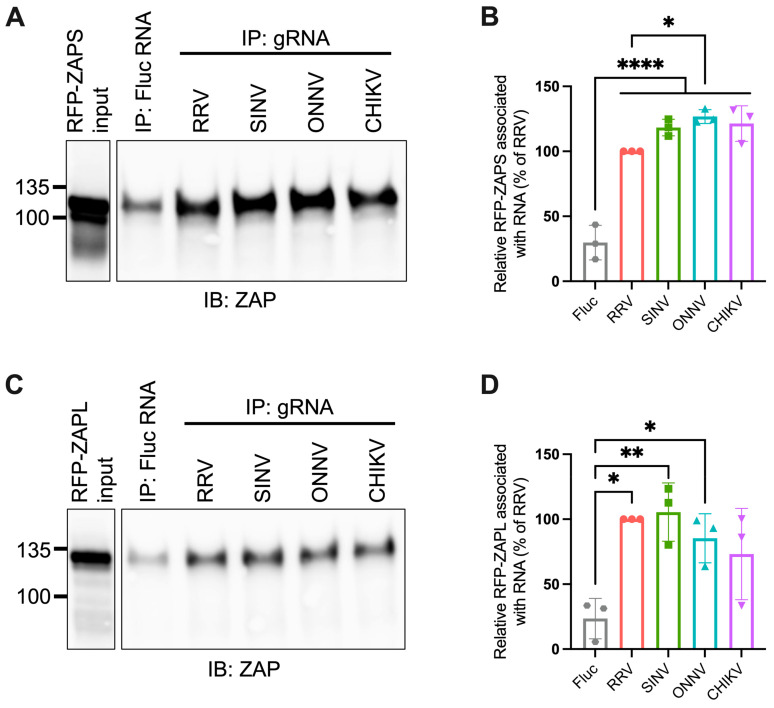
ZAPS and ZAPL binding to alphavirus genomic RNA do not correlate with alphavirus replication sensitivity to ZAP. (**A,C**) Lysates of ZAP KO 293T cells with induced expression of RFP-ZAPS (**A**) or RFP-ZAPL (**C**) were incubated with biotinylated Fluc RNA or gRNA of RRV, SINV, ONNV, or CHIKV. Following streptavidin-mediated pull-down of biotinylated RNA, ZAP associated with each RNA and in whole cell lysate (input) was assayed by immunoblot (IB). Expected sizes: RFP-ZAPS = 106 kDa, RFP-ZAPL = 130 kDa. Data are representative of results from three independent experiments. (**B**,**D**) ImageJ quantifications of panel A (**B**) or C (**D**). Data are combined from three independent experiments. Error bars represent the SD. Asterisks indicate statistically significant differences (one-way ANOVA and Tukey’s multiple comparisons test: *, *p* < 0.05; **, *p* < 0.01; ****, *p* < 0.0001).

Given the importance of ZAP’s ability to bind viral RNA in its inhibition of virus replication, we hypothesized that ZAP-resistant alphaviruses may be able to evade ZAP recognition. Thus, we anticipated there would be greater ZAP binding to the RNA of alphaviruses that are more sensitive to ZAP inhibition (RRV, SINV) than to the RNA of those more resistant (ONNV, CHIKV). To evaluate this hypothesis, we performed in vitro RNA pull-down assays in which we incubated lysates of ZAP KO 293T cells with induced expression of RFP-tagged ZAPS or ZAPL with biotinylated genomic RNA (gRNA) of RRV, SINV, ONNV, or CHIKV. We then immunoprecipitated the biotinylated RNA using streptavidin beads and probed for the presence of bound RFP-ZAPS or -ZAPL. As a control for ZAP background binding, we also assayed ZAP binding to biotinylated firefly luciferase (Fluc) RNA. The resultant ZAP immunoblots were quantified by ImageJ, and the intensity of each RNA-associated ZAP band was normalized to RRV gRNA-associated ZAP to account for variation in overall signal intensity across three independent experiments. In general, in comparison to ZAPS, less ZAPL is pulled down with all RNA, most likely a consequence of comparably lower ZAPL expression in the whole cell lysate input (Figure 3A,C). For ZAPS, we found a significant increase in binding to the gRNA of all tested alphaviruses when compared to the Fluc RNA control (Figure 3B). We also observed a slight increase in ZAPS binding to ONNV gRNA compared to RRV gRNA, contrary to the phenotype of RRV being more ZAP-sensitive than ONNV (Figure 3B). We found a significant increase in ZAPL binding to RRV, SINV, and ONNV gRNA compared to the Fluc control, but not for CHIKV gRNA, likely due to variability across experiments (Figure 3D). Otherwise, as with ZAPS, we found no significant differences in ZAPL binding to alphavirus gRNA (Figure 3D). These data do not support a correlation between the ability of ZAP to inhibit alphavirus replication and the level of ZAP binding to the viral gRNA.

### 3.3. The Alphavirus nsP Gene Region Contains the ZAP Sensitivity Determinant

**Figure 4:**
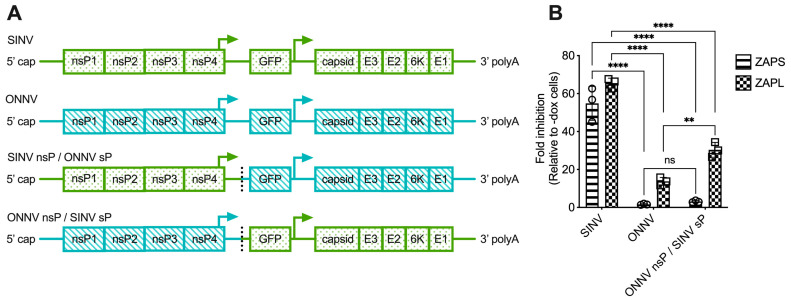
The alphavirus nsP gene region contains the ZAP sensitivity determinant. (**A**) Schematics of chimeric viruses generated. The GFP construct is the same across all viruses. (**B**) ZAP KO 293T cells with doxycycline-inducible expression of ZAPS or ZAPL were infected with GFP-expressing SINV, ONNV, or ONNV nsP/SINV sP chimeric virus at MOI = 0.1 PFU/cell for 18 h before their percentage of infection was determined by flow cytometry. Fold inhibition by ZAP relative to doxycycline-untreated (-dox) cells is shown here. Error bars represent the SD. Data representative of two independent experiments performed with biological replicates in triplicate wells. Asterisks indicate statistically significant differences (two-way ANOVA and Tukey’s multiple comparisons test: **, *p* < 0.01; ****, *p* < 0.0001). ns—not significant.

The lack of correlation between alphavirus ZAP sensitivity and ZAP binding to alphavirus gRNA motivated us to narrow down the alphavirus genome region that confers sensitivity or resistance to ZAP. To this end, we generated chimeric viruses in which we swapped the nsP and sP gene regions of a ZAP-sensitive virus, SINV, and a ZAP-resistant virus, ONNV (Figure 4A). These viruses were chosen because they contain restriction sites that facilitate the ready generation of the chimeric viruses. Additionally, we chose to assay the sensitivity of the chimeric viruses to ZAPS and ZAPL in our ZAP-inducible 293T cell lines, where SINV is significantly more sensitive to ZAP than ONNV [25]. While we were able to generate viable virions of both chimeric viruses from BHK-21 cells, which are defective in IFN production, the SINV nsP/ONNV sP chimeric virus was unable to replicate even in the absence of ZAP in ZAP KO 293T cells, likely due to a more robust IFN response in 293T compared to BHK-21 cells. When we assayed the sensitivity of the ONNV nsP/SINV sP chimeric virus to ZAPS and ZAPL inhibition in the ZAP-inducible ZAP KO 293T cells, we found that its ZAP sensitivity more closely resembles that of ONNV (Figure 4B), demonstrating that the nsP gene region is likely responsible for conferring ZAP sensitivity on an alphavirus.

### 3.4. ZAPS and ZAPL Binding to Alphavirus nsP RNA Do Not Correlate with Alphavirus Replication Sensitivity to ZAP

**Figure 5:**
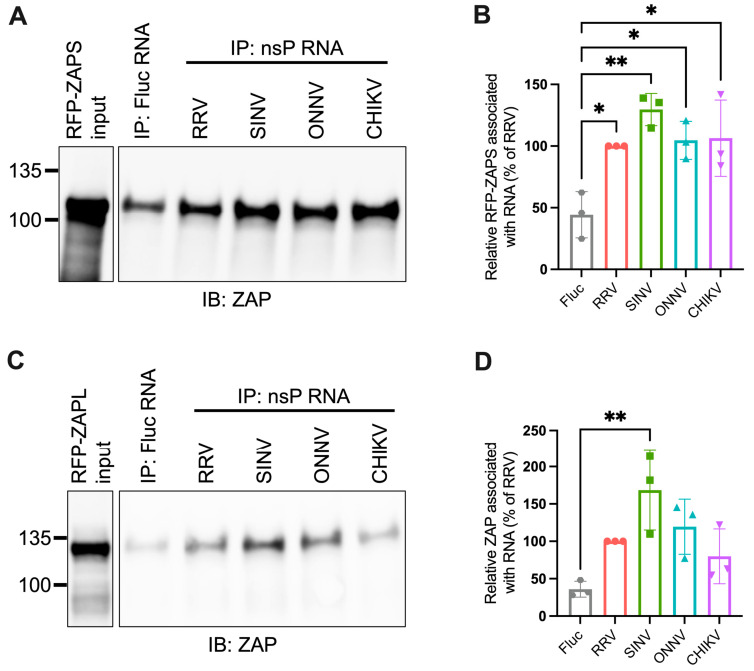
ZAPS and ZAPL binding to alphavirus nsP RNA do not correlate with alphavirus replication sensitivity to ZAP. (**A**,**C**) Lysates of ZAP KO 293T cells with induced expression of RFP-ZAPS (**A**) or RFP-ZAPL (**C**) were incubated with biotinylated Fluc RNA or nsP RNA of RRV, SINV, ONNV, or CHIKV. Following streptavidin-mediated pull-down of biotinylated RNA, ZAP associated with each RNA and in whole cell lysate (input) was assayed by immunoblot. Expected sizes: RFP-ZAPS = 106 kDa, RFP-ZAPL = 130 kDa. Data are representative of results from three independent experiments. (**B,D**) ImageJ quantifications of panel A (**B**) or C (**D**). Data are combined from three independent experiments. Error bars represent the SD. Asterisks indicate statistically significant differences (one-way ANOVA and Tukey’s multiple comparisons test: *, *p* < 0.05; **, *p* < 0.01).

We next used the in vitro RNA binding assay to probe if ZAPS and ZAPL binding to RNA of the nsP region correlates with alphavirus replication sensitivity to ZAP. We found significant enrichment in the binding of ZAPS to all alphavirus nsP RNAs over the Fluc control (Figure 5A,B). However, we found no significant differences when comparing ZAPS binding between different alphavirus nsP RNAs (Figure 5A,B). For ZAPL, we only observed greater enrichment in binding to SINV nsP RNA over the Fluc control (Figure 5C,D). As with ZAPS, there was no significant difference in ZAPL binding between alphavirus nsP RNAs (Figure 5D). We speculate that the increased ZAPL binding to SINV nsP RNA relative to the Fluc control results from the increased levels of CpG dinucleotides in the nsP region of SINV compared to the other alphaviruses [25]. This difference is more apparent with ZAPL than ZAPS, likely because the lower overall expression of ZAPL in the input resulted in less ZAPL pull-down with RNA, allowing us to detect more slight differences in binding. As was the case for gRNA, our results do not support a correlation between the ability of ZAP to inhibit alphavirus replication and the level of ZAP binding to viral nsP RNA.

### 3.5. Sliding Window Analysis of Alphavirus CpG Content Identifies Windows in the nsP Region with CpG Contents Correlating to ZAP Sensitivity

**Figure 6:**
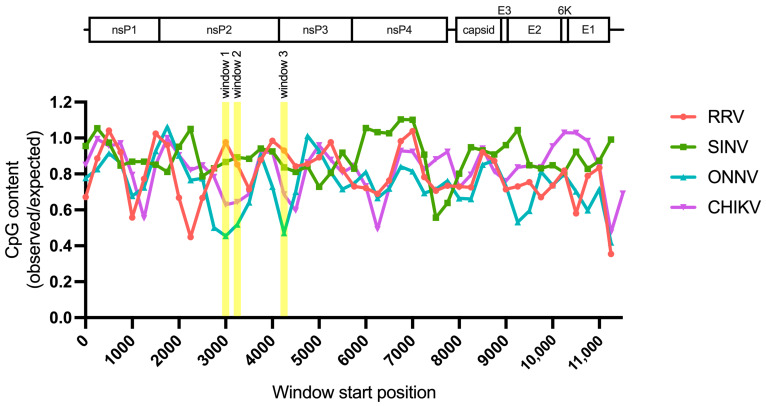
Sliding window analysis of alphavirus CpG content. The ratio of observed CpG dinucleotide frequency to expected frequency based on mononucleotide content (observed/expected) was calculated across alphavirus genomes using a window size of 500-bp and a step size of 250-bp. The genomes were aligned from the start of their 5′ untranslated regions. Windows with CpG contents that correlate with alphavirus replication sensitivity to ZAP are annotated (windows 1–3).

Because we did not find a correlation between alphavirus ZAP sensitivity and ZAP binding to alphavirus nsP RNA, we hypothesized that there may be more localized areas within the nsP region that are more important for ZAP recognition. We decided to look at CpG dinucleotide content to identify the most likely candidate areas, given that ZAP has been demonstrated to selectively bind CpG dinucleotides in HIV-1 RNA, and that CpG content within a specific window of the HIV-1 *env* gene correlates to HIV-1 ZAP sensitivity [31,35]. Since we did not previously identify a correlation between alphavirus CpG content and ZAP replication sensitivity when looking at the nsP region or any specific nsP gene [25], here we took a more targeted approach by calculating CpG content over 500-bp sliding windows across each alphavirus genome with a step size of 250-bp. This analysis revealed three windows where the CpG contents in the genomes of the ZAP-sensitive alphaviruses (RRV, SINV) show a notable enrichment over those in the ZAP-resistant alphaviruses (ONNV, CHIKV): two (windows 1 and 2) in the nsP2 gene, and one (window 3) in the nsP3 gene (Figure 6, Table S4). These windows (henceforth called CpG-correlated windows) are all in the nsP region, consistent with our data demonstrating that the nsP region contains the ZAP sensitivity determinant (Figure 4). Alignments of these window show general nucleotide sequence homology when comparing across alphaviruses (Figure S4). We proceeded with assaying ZAP binding to the RNA of these CpG-correlated windows.

### 3.6. ZAPS and ZAPL Binding to One CpG-Correlated Window Reflects Alphavirus Replication Sensitivity to ZAP and Is CpG-Dependent

**Figure 7:**
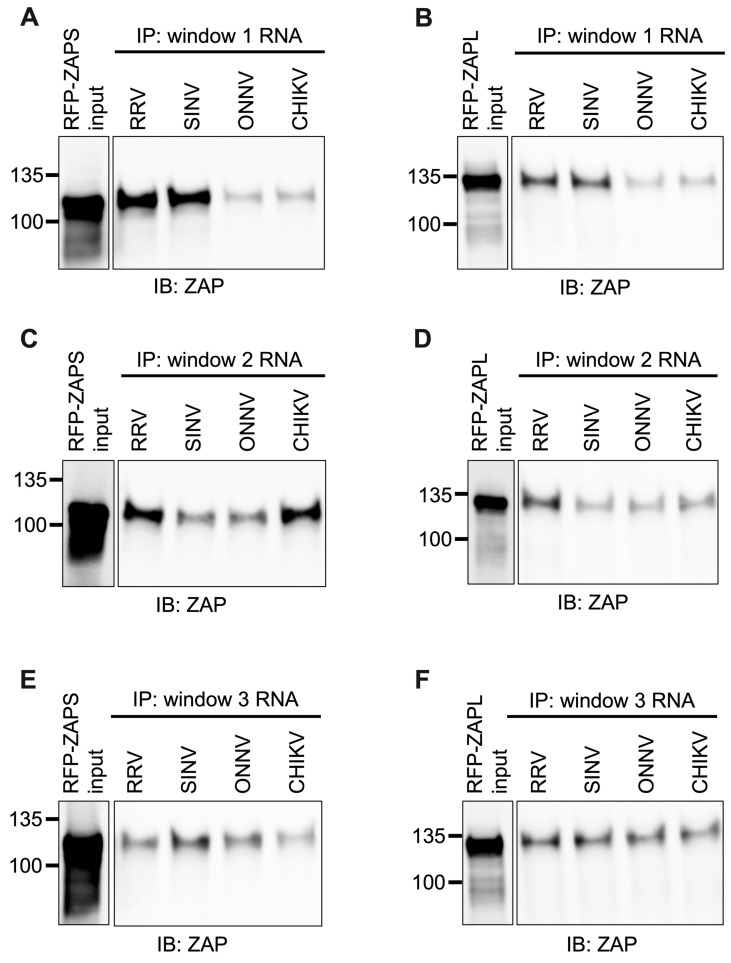
ZAPS and ZAPL binding to one but not all CpG-correlated windows correlates with alphavirus replication sensitivity to ZAP. Lysates of ZAP KO 293T cells with induced expression of RFP-ZAPS (**A**,**C**,**E**) or RFP-ZAPL (**B**,**D**,**F**) were incubated with biotinylated RRV, SINV, ONNV, or CHIKV CpG-correlated window 1 (**A**,**B**), window 2 (**C**,**D**), or window 3 (**E**,**F**) RNA. Following streptavidin-mediated pull-down of biotinylated RNA, ZAP associated with each RNA and in whole cell lysate (input) was assayed by immunoblot. Expected sizes: RFP-ZAPS = 106 kDa, RFP-ZAPL = 130 kDa. Data are representative of results from three independent experiments for window 1 and two independent experiments for windows 2 and 3.

As done for alphavirus gRNA and nsP RNA, we assayed the in vitro ability of ZAPS and ZAPL to bind the RNA of the CpG-correlated windows. We excluded the Fluc RNA background control because it is significantly longer than the windows. As we expected based on CpG content, we observed a clear and significant increase in ZAPS and ZAPL binding to window 1 of the ZAP-sensitive viruses, RRV and SINV, over that of the ZAP-resistant viruses, ONNV and CHIKV (Figure 7A,B and Figure S5A,B), suggesting that differences in alphavirus inhibition may be explained by differential ZAP binding to the viral nsP region in a CpG-dependent manner. However, neither ZAPS nor ZAPL binding to window 2 (Figure 7C,D) or window 3 (Figure 7E,F) correlates with CpG content or ZAP replication sensitivity, indicating that CpG content is not the major determinant for ZAP binding to these windows. Altogether, these results suggest that ZAP may only recognize CpG dinucleotides in specific alphavirus RNA contexts.

**Figure 8:**
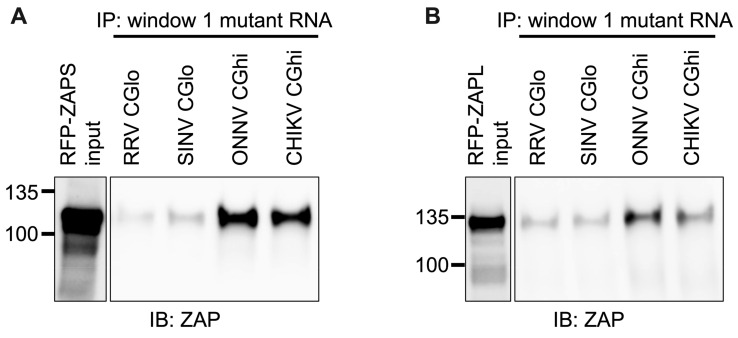
ZAPS and ZAPL binding to CpG-correlated window 1 is CpG-dependent. Lysates of ZAP KO 293T cells with induced expression of RFP-ZAPS (**A**) or RFP-ZAPL (**B**) were incubated with biotinylated RRV CGlo, SINV CGlo, ONNV CGhi, or CHIKV CGhi window 1 mutant RNA. Following streptavidin-mediated pull-down of biotinylated RNA, ZAP associated with each RNA and in whole cell lysate (input) was assayed by immunoblot. Expected sizes: RFP-ZAPS = 106 kDa, RFP-ZAPL = 130 kDa. Data are representative of results from three independent experiments.

We next wanted to confirm that ZAP binding to window 1 is CpG-dependent. To this end, we generated mutants in which we depleted CpG content for the ZAP-sensitive viruses (referred to as CGlo mutants), reducing the observed/expected CpG contents of RRV window 1 from 0.970 to 0.357 and SINV window 1 from 0.864 to 0.446 (Table S4). At the same time, we generated mutants in which we enriched CpG content for the ZAP-resistant viruses (referred to as CGhi mutants), increasing the CpG contents of ONNV window 1 from 0.455 to 1.285 and CHIKV window 1 from 0.626 to 1.437 (Table S4). When we assayed ZAPS and ZAPL binding to these mutants, we found that binding changes in accordance with the change in CpG content: both ZAPS (Figure 8A and Figure S5C) and ZAPL (Figure 8B and Figure S5D) bind less to the CGlo mutants and more to the CGhi mutants compared with unmutated window 1 (Figure 7A,B). The difference in ZAP binding to the CGlo and CGhi mutants is dramatic (about 7-fold for ZAPS and 3-fold for ZAPL), while also significant for ZAPL (Figure S5C,D). Taken together, our results indicate that ZAP selectively binds CpG dinucleotides in the alphavirus nsP region, but this binding is dependent on the context of the CpG dinucleotides within the viral RNA genome.

## 4. Discussion

In this study, we sought to understand the viral determinants of alphavirus sensitivity to the restriction factor ZAP. We first assayed alphavirus sensitivity to endogenous ZAP at baseline and IFN-stimulated expression levels. We found that RRV is most ZAP-sensitive followed by SINV for both viral replication and production, while ONNV replication and production are both ZAP-resistant and CHIKV replication but not production is ZAP-resistant (Figure 1 and Figure 2). We hypothesized that alphaviruses whose replication is more sensitive to ZAP (RRV, SINV) are recognized by ZAP through RNA binding, while alphaviruses more resistant to ZAP (ONNV, CHIKV) evade ZAP binding to their RNA. However, we did not find a correlation between alphavirus replication sensitivity to ZAP and ZAP binding to the genomic (Figure 3) or nsP viral RNA (Figure 5), even though we determined that the ZAP sensitivity determinant mainly lies in the alphavirus nsP region (Figure 4). We then hypothesized that the ZAP sensitivity determinant might be specific to more localized areas of the nsP gene region. Because ZAP is known to bind selectively to CpG dinucleotides, we identified three windows in alphavirus nsP regions with CpG contents correlating to ZAP viral inhibition (Figure 6), and subsequently observed a correlation between binding and sensitivity for one out of the three windows (Figure 7). We confirmed by mutagenesis that ZAP binding to this window in the nsP2 gene is CpG-dependent (Figure 8). Our findings suggest that certain virulent alphaviruses may resist ZAP inhibition by suppressing and/or masking CpG dinucleotide content in specific regions of their RNA genomes, allowing them to escape ZAP recognition.

When studying alphavirus restriction by endogenous ZAP, we found a similar pattern of replication sensitivity to ZAP as we observed with overexpressed individual ZAP isoforms: RRV replication is most sensitive to ZAP inhibition, while SINV replication displays a more intermediate phenotype, and ONNV and CHIKV replication are relatively resistant to inhibition by ZAP (Figure 1). Interestingly, SINV replication appears to be more ZAP-sensitive with IFN pre-treatment than with basal ZAP expression, suggesting that the more IFN-induced ZAP isoforms (ZAPS and the medium ZAP isoform, ZAPM) might have a greater contribution to the restriction of SINV replication. This contrasts our previous result of overexpressed ZAPL and the extralong ZAP isoform being more inhibitory against SINV replication than ZAPS and ZAPM. Studies in which each ZAP isoform is individually depleted in an endogenous context are needed to reconcile these results. Also unexpected was our finding that the CHIKV vaccine strain production is more sensitive to ZAP inhibition than RRV and SINV production (Figure 2), in contrast to CHIKV production being resistant to the ZAP isoforms overexpressed individually [25]. We speculate that this CHIKV production sensitivity to endogenous ZAP results from the synergistic activity of different ZAP isoforms. ZAPS and ZAPL have been demonstrated to play distinct roles in resolution of the IFN response and antiviral activity, respectively, through differential subcellular localization, host mRNA binding, and expression at basal versus IFN-induced conditions [28,47]. It is possible that the coordinated effects of these distinct functions may result in the inhibition of CHIKV production. Additionally, ZAP can multimerize with itself through the N-terminus shared by all isoforms [48], so it is possible that the different ZAP isoforms can directly interact to mediate functions they do not perform individually. Further work is needed to determine the mechanism by which endogenous ZAP can inhibit CHIKV production, as well as the CHIKV life cycle step between genome replication and virion production that is acted upon by ZAP or another ISG dependent on ZAP for its antiviral activity. This would represent a novel mechanism for alphavirus inhibition by ZAP, as previous work in SINV showed that ZAP blocks the early step of incoming viral genome translation [20]. Studies with an unattenuated, non-vaccine strain of CHIKV are also needed to evaluate if the effect of ZAP on CHIKV production also occurs for a more clinically relevant strain of CHIKV.

We did not find a correlation between ZAP binding to genomic (Figure 3) or nsP (Figure 5) alphavirus RNA and alphavirus replication sensitivity to ZAP, but we did observe such a correlation for one specific window of the nsP2 gene with CpG content correlating to sensitivity (Figure 7). We do acknowledge that this correlation does not necessarily demonstrate that ZAP binding to this region has functional importance. Further studies are needed in which this region is mutated within the alphavirus genomes to modulate ZAP binding, which would allow for a more definitive link between ZAP binding and inhibition of alphavirus replication to be made. As it is, our results here are consistent with the finding that CpG content in only one specific region of the HIV-1 *env* gene correlates with HIV-1 strain sensitivity to ZAP [35]. It also remains unclear why ZAP binding to a specific alphavirus RNA region would correlate with ZAP sensitivity rather than overall ZAP binding to the viral RNA, particularly in the context of translation inhibition by ZAP. One possibility is that this region may contain a *cis*-acting signal important in alphavirus genome translation, and that ZAP binding may shield or alter the signal. For example, ZAP was recently shown to block ribosomal frameshifting in SARS-CoV-2 by interfering with folding of an RNA element [49]. ZAP binding may also interfere with a secondary structure important for the translation of the ZAP-sensitive alphaviruses. Chemical probing of alphavirus RNA shows that alphavirus RNA structures are highly divergent [50,51], making it difficult to identify structures that may play a functional role based on conservation. Future experiments in which this nsP2 window is mutated to independently alter its sequence or secondary structure are needed to evaluate its role in alphavirus translation and ZAP sensitivity. Furthermore, given our targeted approach of identifying regions potentially important for ZAP inhibition by CpG content correlation, future research should also include a more comprehensive study of the regions required for ZAP binding and inhibition within each alphavirus RNA.

Despite ZAP’s known function as a CpG dinucleotide sensor, ZAP binding only correlates with CpG content for one of our tested windows (Figure 7 and Figure 8). Our findings suggest that CpG content is not the only determinant for ZAP binding to alphavirus RNA, or that CpG content in only particular regions may sensitize an alphavirus to ZAP recognition and inhibition. ZAP has also been demonstrated to recognize UpA motifs, but we did not find a correlation between UpA content and ZAP binding to our tested windows (Table S4). ZAP may be recognizing additional motifs that have yet to be characterized. Alternatively, ZAP may indeed be binding CpG dinucleotides, but only when they are in an accessible conformation: thus, for CpG-correlated window 1, both CpG content and accessibility correlate with ZAP binding and inhibition, while only CpG content but not accessibility correlate for the remaining windows. Future studies combining RNA structure prediction programs with experimental methods of structure characterization are needed to determine the folding patterns of our tested windows, as well as how these patterns may be altered within the context of full-length viral RNA and in cellular contexts. Further studies are also required to interrogate the exact alphavirus sequence and structural elements recognized by ZAP, and if any of these elements are shared among different viruses inhibited by ZAP.

Some viruses actively antagonize ZAP by interfering with its function or expression. While here we focused on the role of ZAP RNA binding in sensitizing alphaviruses to ZAP, future studies should also address the possibility that ZAP-resistant alphaviruses may possess ZAP antagonism activity. The ONNV nsP/SINV sP chimeric virus not only delivers and replicates a chimeric viral RNA sequence during infection but also produces chimeric viral proteins (Figure 4), so it is possible that the ZAP resistance phenotype of this chimeric virus results from an ONNV nsP product antagonizing ZAP. Additional experiments are needed to evaluate this possibility. Examples of other viruses antagonizing ZAP include influenza A virus preventing ZAP from binding to its target RNA [21], herpes simplex virus 1 degrading ZAP mRNA [22], and enterovirus A71 cleaving ZAP protein [23]. These known mechanisms of ZAP antagonism provide starting points for investigating potential alphavirus ZAP antagonism.

ZAP-resistant alphaviruses may also evade ZAP inhibition through more indirect means, such as altering ZAP’s ability to interact with its co-factors. ZAP inhibition of alphaviruses requires its ability to interact with the E3 ubiquitin ligase TRIM25, as well as the ligase activity of TRIM25 [10]. Thus, it would be interesting to probe if infection with ZAP-resistant alphaviruses may modulate ZAP’s ability to interact with TRIM25 or TRIM25’s ability to ubiquitinate its target proteins and mediate antiviral activity [52]. Given that ZAP has been demonstrated to bind and regulate the expression of host mRNAs [31,47,53,54], it is also possible that ZAP-resistant alphaviruses may alter ZAP binding to host mRNA targets that encode additional ZAP co-factors or alter the host antiviral state more generally. This latter possibility is especially intriguing, given the role of ZAPS in regulating the IFN response [28]. To evaluate these hypotheses, studies are needed to characterize changes in ZAP binding to host mRNA targets and their expression upon infection with ZAP-sensitive or -resistant alphaviruses.

In conclusion, our work provides insight into a potential alphavirus virulence strategy of innate immune evasion. Given the re-emergence and spread of highly pathogenic alphaviruses like CHIKV, our study is critical and timely for understanding the determinants of alphavirus virulence, which will provide molecular targets for the development of attenuated vaccines and therapies to reinforce weak points of the immune response.

## Data Availability

The data presented in this study are available on request from the corresponding author.

## Supplementary Materials

The following supporting information can be downloaded at: https://www.mdpi.com/article/10.3390/v15040830/s1, Figure S1: Streptavidin-HRP dot blots of biotinylated RNA; Figure S2: Pre-treatment with WT and ZAP KO 293T cells with IFN prior to SINV infection; Figure S3: Alphavirus production shows differential sensitivity to basal endogenous ZAP in 293T cells; Figure S4: Alignments of CpG-correlated windows; Figure S5: Quantifications of ZAPS and ZAPL binding to CpG-correlated window 1 RNA and CpG-correlated window 1 mutant RNA; Table S1: Primers for generation of EGFP-expressing CHIKV vaccine strain 181/clone 25; Table S2: Primers to generate CpG-correlated window DNA templates for T7 transcription; Table S3: qPCR primers; Table S4: CpG and UpA dinucleotide contents of CpG-correlated windows.
