# Supplementary material for: Alphavirus Evasion of Zinc Finger Antiviral Protein (ZAP) Correlates with CpG Suppression in a Specific Viral nsP2 Gene Sequence
Source: Viruses. 2023 Mar 24;15(4):830. doi: 10.3390/v15040830 (PMC10145277; doi:10.3390/v15040830)
Supplement: Supplementary file 1 [file viruses-15-00830-s001.zip › viruses-2292108-supplementary.pdf]

Supplementary Figures

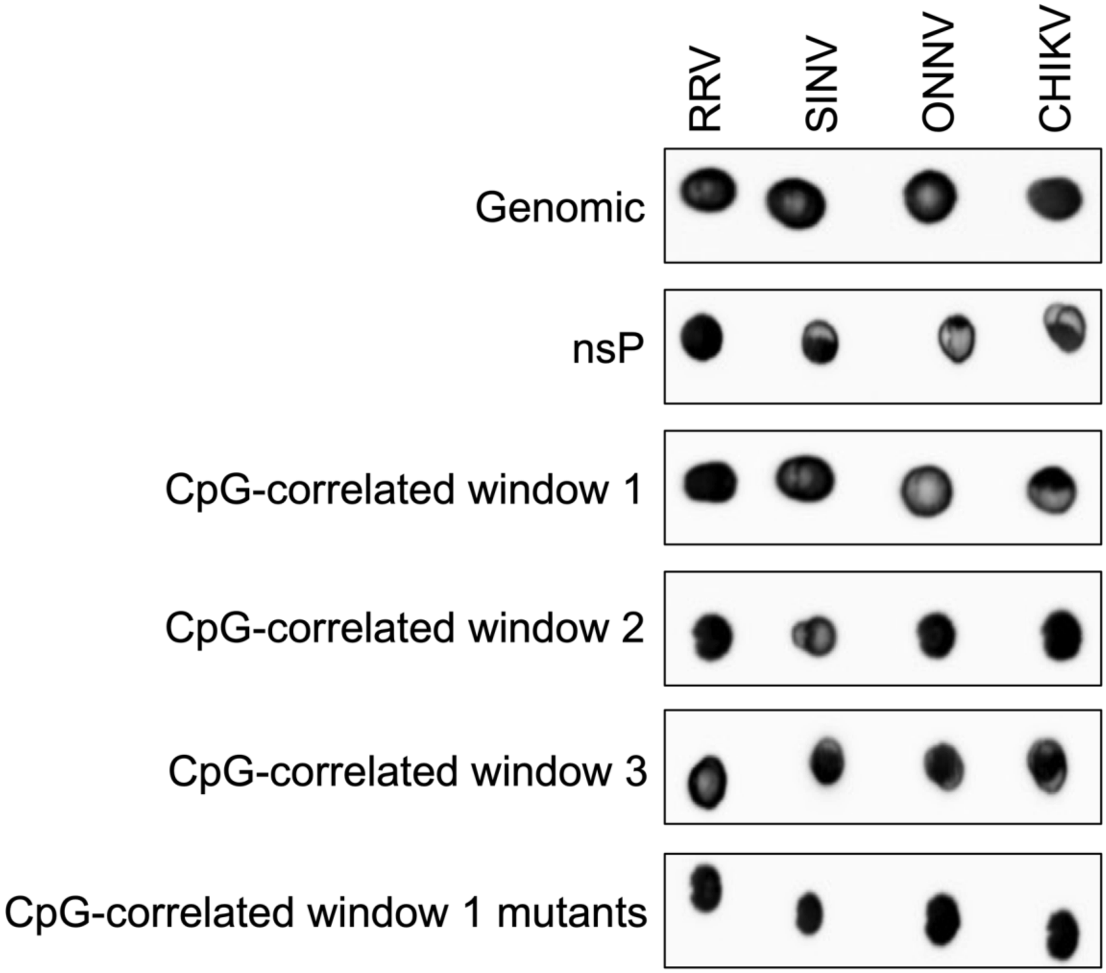

Figure S1. Streptavidin-HRP dot blots of biotinylated RNA. RNA shown in this figure were used for RNA immunoprecipitation shown in Figures 3, 5, 7, and 8.

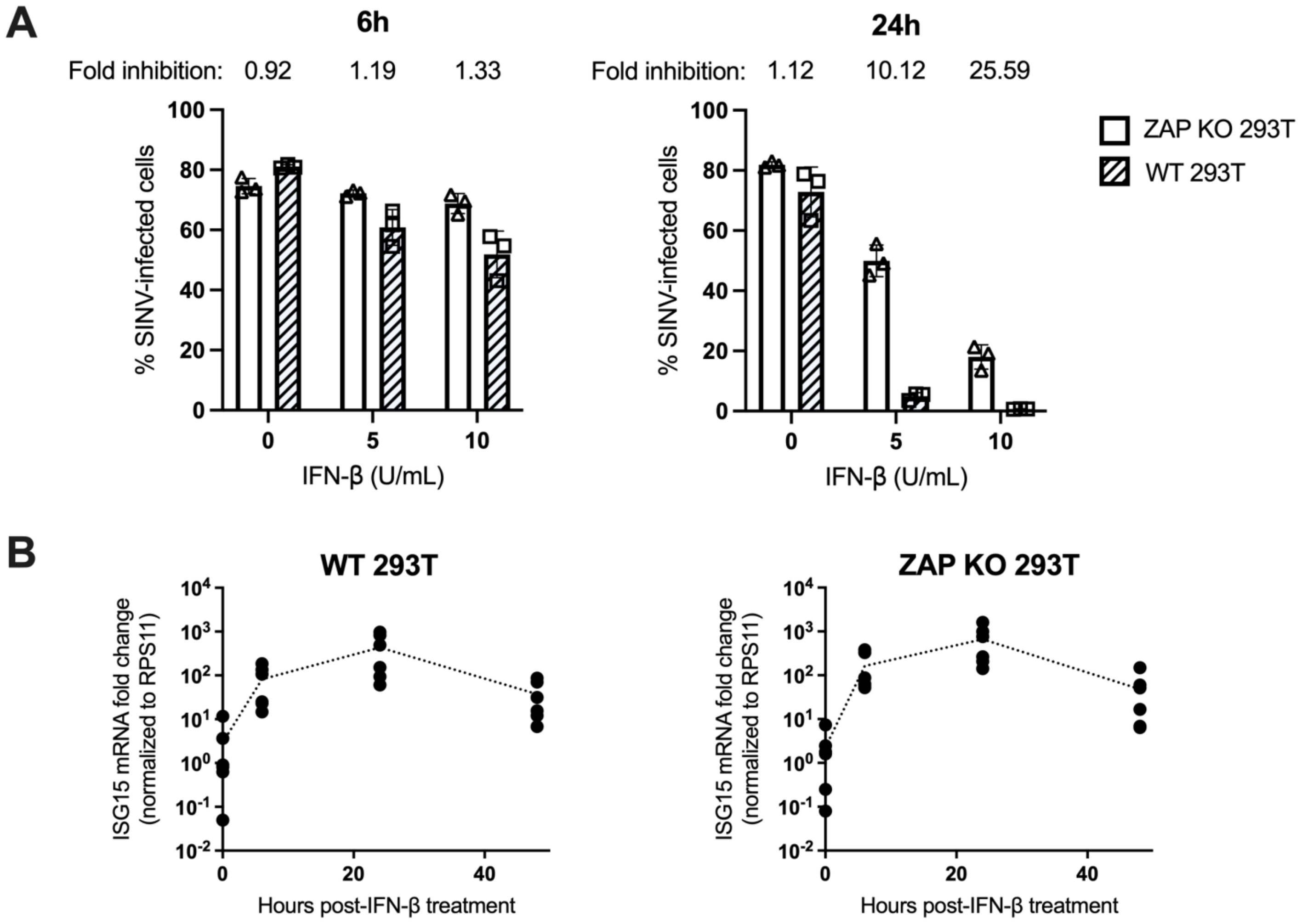

Figure S2. Pre-treatment of WT and ZAP KO 293T cells with IFN prior to SINV infection. (**A**) Following treatment with 0, 5, or 10 U/mL IFN-β for 6 or 24 h, ZAP KO and WT 293T cells were infected with GFP-expressing SINV (MOI = 1 PFU/cell). Cells were harvested and fixed at 24 h.p.i., and their percent infection was determined by flow cytometry. Fold inhibition of SINV in WT 293T was calculated relative to ZAP KO 293T for each IFN-β treatment condition to indicate the antiviral effects of endogenous ZAP. (**B**) WT and ZAP KO 293T cells were treated with 5 U/mL IFN-β, and total RNA was extracted at 0, 6, 24, and 48 h post-IFN-treatment for RT-qPCR analysis of ISG15 and RPS11 mRNA levels.

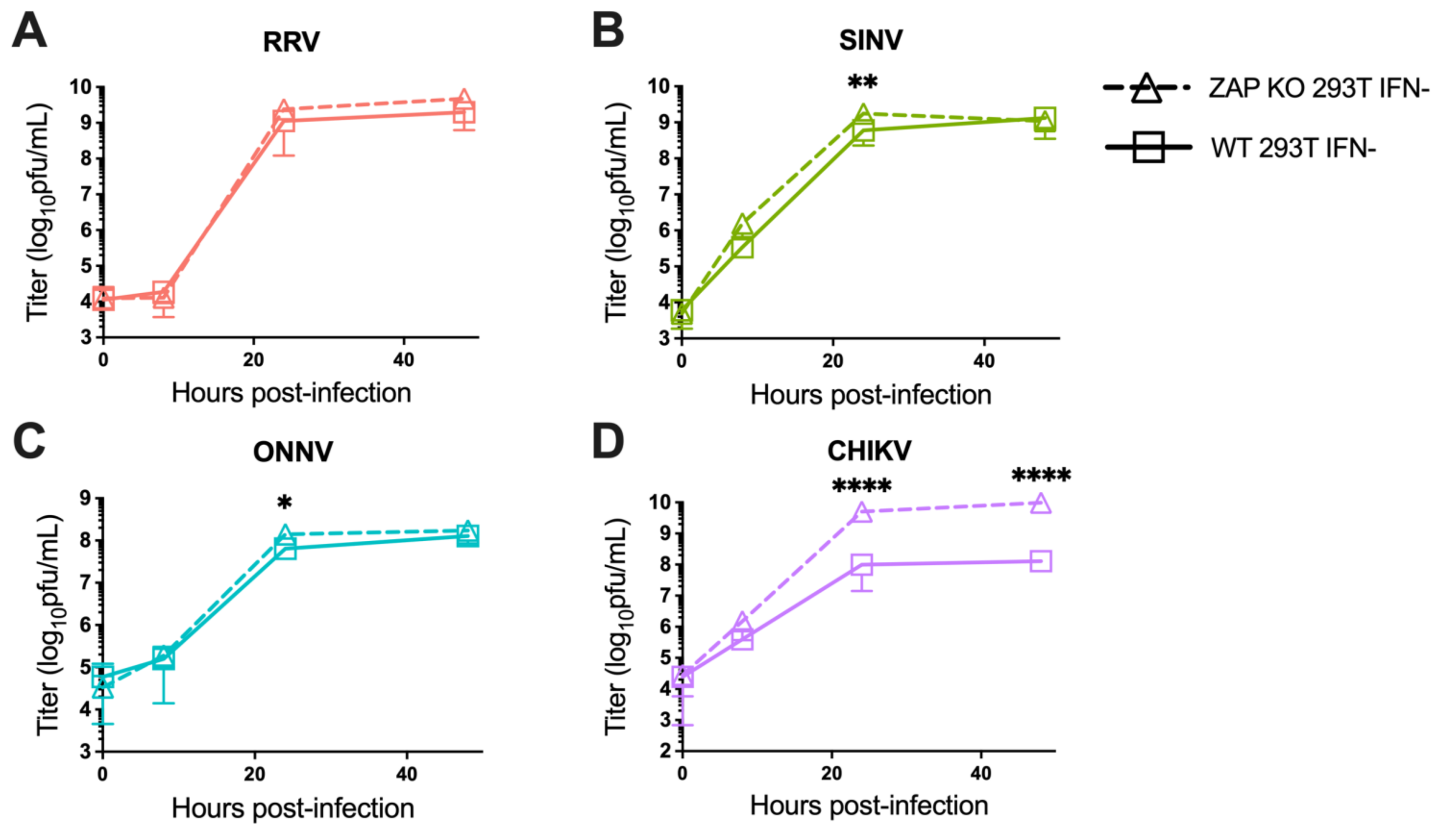

Figure S3. Alphavirus production shows differential sensitivity to basal endogenous ZAP in 293T cells. ZAP KO and WT 293T untreated with IFN-β were infected with RRV (**A**), SINV (**B**), ONNV (**C**), and the CHIKV vaccine strain (181/clone 25) (**D**) at MOI = 0.1 PFU/cell. Media overlaying the cells were harvested at 0, 8, 24, and 48 h.p.i., and the viral titer was determined by infection of BHK-21 cells in standard plaque assays. Mean values from four biological replicates across two independent experiments are plotted, and error bars represent the SD. Asterisks indicate statistically significant differences (two-way ANOVA with Bonferroni posttest: *, $p < 0.05$; **, $p < 0.01$; ****, $p < 0.0001$).

Window 1

```
SINV_window_1       CAUACCUAAAGGAAACUUUCAGGCUACUAUAGAGGACUGGGAAGCUGAACACAAGGGAAU
RRV_window_1        ---------GGGCGAUUUCAGCGCUACGUUGGAGGAGUGGCAAGAGGAGCAUGACAACAU
ONNV_window_1       -----CAAAAGGGAACUUUAAGGCAACAAUCAAGGAGUGGGAAGCGGAACACGCCUCCAU
CHIKV_window_1      ----------GGAAACUUCAAAGCAACUAUUAAGGAGUGGGAGGUGGAGCACGCAUCGAU
                              **  * **    ** **  *  **** *** * *  ** **       **

SINV_window_1       AAUUGCUGCAAUAAACAGCCCCACUCCCCGUGCCAAUCCGUUCAGCUGCAAGACCAACGU
RRV_window_1        CAUGAACGCCCUUCGCGAGAGGUCGACAGCAGUAGACCCGUUCCAGAACAAAGCCAAAGU
ONNV_window_1       UAUGGCAGGAAUAUGCAAUCACCAGAUGGCUUUUGACACAUUUCAGAACAAAGCUAAUGU
CHIKV_window_1      AAUGGCGGGCAUCUGCAGUCACCAAGUGACCUUUGACACAUUCCAAAACAAAGCCAACGU
                     **    *   *   *                   *  * **      ***  * ** **

SINV_window_1       UUGCUGGGCGAAAGCAUUGGAACCGAUACUAGCCACGGCCGGUAUCGUACUUACCGGUUG
RRV_window_1        CUGCUGGGCAAAGUGCCUCGUGCAGGUCCUAGAAACGGCUGGGAUACGCAUGACGGCAGA
ONNV_window_1       AUGCUGGGCUAAAUGCCUGGUCCCUAUUCUUGACACCGCUGGAAUCAAACUAAGUGACAG
CHIKV_window_1      UUGCUGGGCUAAGAGCUUGGUCCCUAUCCUCGAAACAGCGGGGAUAAAACUAAAUGAUAG
                     ******** **     * *  *   * ** *  ** ** ** **     * *  *

SINV_window_1       CCAGUGGAGCGAACUGUUCCCACAGUUUGCGGAUGACAAACCACAUUCGGCCAUUUACGC
RRV_window_1        GGAGU---GGGAUACAGUGUUGGCUUUCCGCGAAGACAGGGCGUACUCACCCGAAGUGGC
ONNV_window_1       GCAGUGGUCUCAGAUAGUGCAAGCUUUUAAAGAAGAUAGGGCCUACUCUCCAGAAGUUGC
CHIKV_window_1      GCAGUGGUCCCAGAUAAUUCAAGCCUUCAAAGAAGACAAAGCAUACUCACCCGAAGUAGC
                      ***      *     *       **    ** ** *   *  * **  *       **

SINV_window_1       CUUAGACGUAAUUUGCAUUAAGUUUUUCGGCAUGGACUUGACAAGCGGACUGUUUUCUAA
RRV_window_1        UCUGAACGAGAUCUGUACCAAGUAUUACGGCGUUGACUUAGACAGCGGAUUGUUCUCCGC
ONNV_window_1       ACUGAAUGAAAUAUGUACCCGCAUAUAUGGGGUAGAUCUGGACAGCGGACUAUUUUCAAA
CHIKV_window_1      CCUGAAUGAAAUAUGCACGCGCAUGUAUGGGGUGGAUCUAGACAGUGGGCUAUUCUCUAA
                      *  * *  ** ** *        *  **  * **  *    ** **  * ** **

SINV_window_1       ACAGAGCAUCCCACUAACGUACCAUCCCGCCGAUUCAGCGAGGCCGGUAGCUCAUUGGGA
RRV_window_1        CCAAUCGGUCUCAUU---GUACUAUGA-----------------AAAC-AACCACUGGGA
ONNV_window_1       GCCGCUGAUAUCCGU---CUACUAUGC-----------------AGAC-AACCACUGGGA
CHIKV_window_1      ACCGUUGGUAUCUGU---GUAUUACGC-----------------GGAU-AACCAUUGGGA
                     *      *  *  *    **  *                            ** *****

SINV_window_1       CAACAGCCCAGGAACCCGCAAGUAUGGGUACGAUCACGCCAUUGCCGCCGAACUCUCCCG
RRV_window_1        CAAUAGACCGGGCGGCCGGAUGUAUGGAUUCAACCGCGAAGUCGCCCGUAAGUUUGAGCA
ONNV_window_1       CAAUAGACCAGGAGGAAAAAUGUUCGGGUUCAACCCUGAGGUGGCACUUAUGCUUGAAAA
CHIKV_window_1      UAAUAGGCCGGGAGGAAAGAUGUUCGGAUUCAACCCUGAGGCAGCGUCCAUUCUAGAAAG
                     ** ** ** **       * **  ** * * * *  *     **        *

SINV_window_1       UAGAUUUCCGGUG-UUCCAGCUAGCUGGGA--AGGGCACACAACUUGAUUUGCAGA-CGG
RRV_window_1        ACGCUACCCAUUCCUGAGAGGCAAGAUGGACUCGGGGCUACAAGUUAAUGU----UCCAG
ONNV_window_1       GAAAUAUCCCUUUACAAAAGGUAAGUGGAACAU----CAACAAGCAGAUAUGUAUAACUA
CHIKV_window_1      AAAGUACCCAUUUACAAAAGGAAAGUGGAACAU----CAACAAGCAGAUCUGCGUGACUA
                        *  **  *      **  *    * *         ****    ** *      *

SINV_window_1       GGAGAACC-----AGAGU-UAUCUCUGC--------------------ACAG-----
RRV_window_1        AGAGAAAAGUACAGCCAUUCAAUGCGGAAUGUAAUAUAUUACCAUCAAACAGGCGAC
ONNV_window_1       CCAGAAAGGUUGACGAAUUUAACCCCGAAACCAACAUAAUACCGGCCAAC-------
CHIKV_window_1      CCAGGAGGAUAGAAGACUUCAACCCUACCACCAACAUUAUACCGGUCAACAGG----
                      ** *           *  *   *                       **
```

Window 2

```
SINV_window_2       UUUGCAUUAAGUUUUUCGGCAUGGACUUGACAAGCGGACUGUUUUCUAAACAGAGCAUCC
RRV_window_2        -----------UAUUACGGCGUUGACUUAGACAGCGGAUUGUUCUCCGCCCAAUCGGUCU
ONNV_window_2       ----UACCCGCAUAUAUGGGGUAGAUCUGGACAGCGGACUAUUUUCAAAGCCGCUGAUAU
CHIKV_window_2      -------GCGCAUGUAUGGGGUGGAUCUAGACAGUGGGCUAUUCUCUAAACCGUUGGUAU
                                  *  **  * **  *    ** **  * ** **    *      *

SINV_window_2       CACUAACGUACCAUCCCGCCGAUUCAGCGAGGCCGGUAGCUCAUUGGGACAACAGCCCAG
RRV_window_2        CAUU---GUACUAUGA-----------------AAAC-AACCACUGGGACAAUAGACCGG
ONNV_window_2       CCGU---CUACUAUGC-----------------AGAC-AACCACUGGGACAAUAGACCAG
CHIKV_window_2      CUGU---GUAUUACGC-----------------GGAU-AACCAUUGGGAUAAUAGGCCGG
                    *  *    **  *                            ** ***** ** ** ** *

SINV_window_2       GAACCCGCAAGUAUGGGUACGAUCACGCCAUUGCCGCCGAACUCUCCCGUAGAUUUCCGG
RRV_window_2        GCGGCCGGAUGUAUGGAUUCAACCGCGAAGUCGCCCGUAAGUUUGAGCAACGCUACCCAU
ONNV_window_2       GAGGAAAAAUGUUCGGGUUCAACCCUGAGGUGGCACUUAUGCUUGAAAAGAAAUAUCCCU
CHIKV_window_2      GAGGAAAGAUGUUCGGAUUCAACCCUGAGGCAGCGUCCAUUCUAGAAAGAAAGUACCCAU
                    *       * **  ** * * * *  *     **        *          *  **

SINV_window_2       UG-UUCCAGCUAGCUGGGA--AGGGCACACAACUUGAUUUGCAG-ACGGGGAGAAC----
RRV_window_2        UCCUGAGAGGCAAGAUGGACUCGGGGCUACAAGUUAAUGU----UCCAGAGAGAAAAGUA
ONNV_window_2       UUACAAAAGGUAAGUGGAACAU----CAACAAGCAGAUAUGUAUAACUACCAGAAAGGUU
CHIKV_window_2      UUACAAAAGGAAAGUGGAACAU----CAACAAGCAGAUCUGCGUGACUACCAGGAGGAUA
                    *      **  *    * *         ****    ** *      *    ** *

SINV_window_2       -CAGAGUUAUCUCUGCACAGCAUAACCUGGUCCCGGUGAACCGCAAUCUUCCUCACGCCU
RRV_window_2        CAGCCAUUC--AAUGCGGAAUGUAAUAUAUUACCAUCAAACAGGCGACUUCCGCACGCCC
ONNV_window_2       GACGAAUUU--AACCCCGAAACCAACAUAAUACCGGCCAACCGAAGACUGCCGCACUCAC
CHIKV_window_2      GAAGACUUC--AACCCUACCACCAACAUUAUACCGGUCAACAGGAGACUACCACACUCAU
                          **       *       **  *  * **    *** *    ** ** *** *

SINV_window_2       UAGUCCCCGAGUACAAG-GAGAAGCAACCCGGCCCGGUCAAAAAAUUCUUGAACCAGUUC
RRV_window_2        UCGUCACCAGCUACCAG-CAGUGCCAGGGCGAGAGGGUAGAAUGGCUUUUGAAGAAGCUU
ONNV_window_2       UCGUGGCUGAACACCAUACAGUGAGA-GGGGAAAGAAUGGAAUGGCUGGUAAACAAAAUC
CHIKV_window_2      UAGUGGCCGAACACCGCCCAGUAAAA-GGGGAAAGAAUGGAAUGGCUGGUUAACAAGAUA
                    * **  *    **      **    *    *      * **     *  * **  *  *

SINV_window_2       AAACACCACUCAGUACUUGUGGUAUCAGAGGAAAAAAUUGAAGCUCCCCGUAAGAGAAUC
RRV_window_2        CCCGGAUACCAUUUAUUGCUGGUAAGCGAGUACAAUCUGGCGCUGCCCCAUAAAAGAGUC
ONNV_window_2       AACGGUCACCACAUGUUGUUGGUUAGUGGUUAUAAUCUUAUAUUACCAACAAAAAGAGUC
CHIKV_window_2      AACGGACACCACGUACUCCUGGUUAGCGGCUAUAACCUUGCACUGCCUACUAAGAGAGUC
                           **    *  *  ****    *   * **  *       **    ** *** **

SINV_window_2       GAAUGGAUCGCCCCGAUUGGCAUAGCCGGUGCAGAUAAGAACUACAACCUGGCUUUCGGG
RRV_window_2        UUUUGGAUUGCACCACCCCAUGUGUCUGGUGCAGAUCGUAUUUAUGAUCUUGACCUAGGA
ONNV_window_2       ACCUGGGUAGCACCGUUAGGCACCAGAGGUGCAGACUACACAUAUAACCUGGAACUUGGU
CHIKV_window_2      ACCUGGGUAGCGCCACUAGGUGUCCGCGGAGCGGACUAUACAUACAACCUAGAGCUGGGU
                       *** * ** **             ** ** **    *  **  * ** *   * **

SINV_window_2       UUUCCGCCGCAG---GCACGGUACGACCUGGUG--------------------------
RRV_window_2        UUACCCCUGAAUGCAGGCCGUUACGACUUGGUAUUUGUGAACAUACACACUGAGUACAG
ONNV_window_2       CUACCAGCCACACUAGGCAGAUAUGACCUGGUAGUUAUCAAUAUCCAUACUC-------
CHIKV_window_2      CUACCAGCAACGCUUGGUAGGUAUGACCUAGUGGUCAUAAACAUCCACACACCUU----
                     * **          *   * ** *** * **
```

Window 3

```
SINV_window_3       GGAGACAGGCACCGCAAGAAUGACUGUGUGCCUAGGAAAGAAAGUGAUCCACGCGGUCGG
RRV_window_3        -----------GGCCA------A-------CGGUAUGA---AUGUCAUCCACGCGGUAGG
ONNV_window_3       --------------CA------AUCAUGUGCGGUCAAUACCCCGUCAUCCACGCGGUAGG
CHIKV_window_3      ---------------------------GUGCGGUACGUAUCCAGUAAUCCACGCCGUAGG
                                                  *            ** ******** ** **

SINV_window_3       CCCUGAUUUCCGGAAGCACCCAGAAGCAGAAGCCUU----GAAAUUGCUACAAAACGCCU
RRV_window_3        CCCGAAUUUCUCCACGGUGACCGAGGCAGAGGGCGACAGAGAGUUGGCC----GCCGCAU
ONNV_window_3       CCCAAACUUCUCAAACUAUUCUGAGGCUGAAGGGGAUAGGGAAUUGGCU----UCAGUGU
CHIKV_window_3      ACCAAACUUCUCAAAUUAUUCGGAGUCUGAAGGGGACCGGGAAUUGGCG----GCUGCCU
                     **  * ***   *      * **  * ** *        **  * **        *  *

SINV_window_3       ACCAUGCAGUGGCAGACUUAGUAAAU--GAACAUAACAUCAAGUCUGUCGCCAUUCCACU
RRV_window_3        ACCGUGCCGUGGCG--GGUAUUAUCAAUGCUAGUAACAUUAAGAGUGUAGCCAUCCCUCU
ONNV_window_3       AUAGAGAAGUGGCGAAAGAAGUGUCUAGGCUAGGAG--UGAGCAGUGUAGCCAUCCCUUU
CHIKV_window_3      AUCGAGAAGUCGCAAAGGAAGUAACUAGACUGGGAG--UAAAUAGCGUAGCUAUACCUCU
                    *    *  ** **      * *            *   * *     ** ** ** **  *

SINV_window_3       GCUAUCUACAGGCAUUUACGCAGCCGGAAAAGACCGCCUUGAAGUAUCACUUAACUGCUU
RRV_window_3        GUUGUCGACGGGAGUGUUCUCCGGAGGUAAAGAUAGAGUCAUGCAGUCACUAAAUCACCU
ONNV_window_3       GCUUUCAACCGGUGUGUACUCAGGAGGCAAAGAUAGACUGCUGCAAUCACUAAACCAUCU
CHIKV_window_3      CCUCUCCACAGGUGUAUACUCAGGAGGGAAAGACAGGCUAACCCAGUCACUGAACCACCU
                      * ** ** **  * * * * *  ** *****  *  *       ***** **     *

SINV_window_3       GACAACCGCGCUAGACAGAACUGACGCGGACGUAACCAUCUAUUGCCUGGAUAAGAAGUG
RRV_window_3        GUUUACCGCAAUGGACACCACGGACGCUGACGUAGUCAUCUAUUGCCGCGACAAAGCCUG
ONNV_window_3       UUUCACAGCGAUGGAUUCGACAGAUGCAGAUGUUGUCAUCUAUUGCAGGGAUAAGGAAUG
CHIKV_window_3      CUUUACAGCCAUGGACUCGACGGAUGCAGACGUGGUCAUCUACUGCCGAGACAAGGAAUG
                        ** **  * **    ** ** ** ** **   ****** ***   ** **    **

SINV_window_3       GAAGGAAAGAAUCGACGCGGCACUCCAACUUAAGGAGUCUGUAACAGAGCUGAAGGAUGA
RRV_window_3        GGAGAAGAAAAUCCAGGAGGCUAUCGAUCGCCG---CACCGCCGUGGAAUUGGUAUCUGA
ONNV_window_3       GGAGAAGAAGAUCACUGAAGCCAUAUCAUUAAG---AUCCCAGGUAGAACUACUAGAUGA
CHIKV_window_3      GGAGAAGAAAAUAUCUGAGGCCAUACAGAUGCG---GACCCAAGUGGAGCUGCUGGAUGA
                    * ** * *  **    *  **  *              *       **  *      ***

SINV_window_3       AGAUAUGGAGAUCGACGAUGAGUUAGUAUGGAUUCAUCCAGACAGUUGCUUGAAGGGAAG
RRV_window_3        AGACAUCUCACUCGAGUCUGACUUGAUACGGGUACACCCAGAUAGUUGCUUGGUAGGCAG
ONNV_window_3       UCACAUCUCAGUGGAUUGCGACAUUGUACGCGUUCAUCCAGACAGCAGCUUGGCAGGCCG
CHIKV_window_3      GCACAUCUCCAUAGACUGCGAUGUCAUUCGCGUGCACCCUGACAGUAGCUUGGCAGGCAG
                      * **     * **    **  *  *  *  * ** ** ** **  *****   **  *

SINV_window_3       AAAGGGAUUCAGUACUACAAAAGGAAAAUUGUAUUCGUACUUCGAAGGCACCAAAUUCCA
RRV_window_3        AAAAGGUUACAGCAUAACAGAUGGGAAGCUGCAUUCAUACCUGGAAGGUACCCGCUUUCA
ONNV_window_3       AAAGGGGUACAGCACAGUAGAGGGAGCACUCUACUCGUACCUAGAGGGAACAAGAUUCCA
CHIKV_window_3      AAAAGGAUACAGCACCACGGAAGGCGCACUGUAUUCAUAUCUAGAAGGGACACGUUUUCA
                    *** ** * *** *      * **     *  * ** **  * ** ** **    ** **

SINV_window_3       UCAAGCAGCAAAAGACAUGGCGGAG------------------------------
RRV_window_3        UCAGACUGCGGUGGACAUGGCUGAGAUAUCUACCUUGUGGCCGAAACUUCAGGAC
ONNV_window_3       CCAAACUGCUGUAGAUAUGGCAGAAAUAUAUACCAUGUGGCCAAAACAAAC----
CHIKV_window_3      CCAGACGGCAGUGGAUAUGGCAGAGAUAUACACUAUGUGGCCAAAGCAAACAGAG
                     **  * **    ** ***** **
```

Figure S4. Alignments of CpG-correlated windows. Sequences of CpG-correlated windows identified in Figure 6 were aligned across alphaviruses using Clustal Omega.

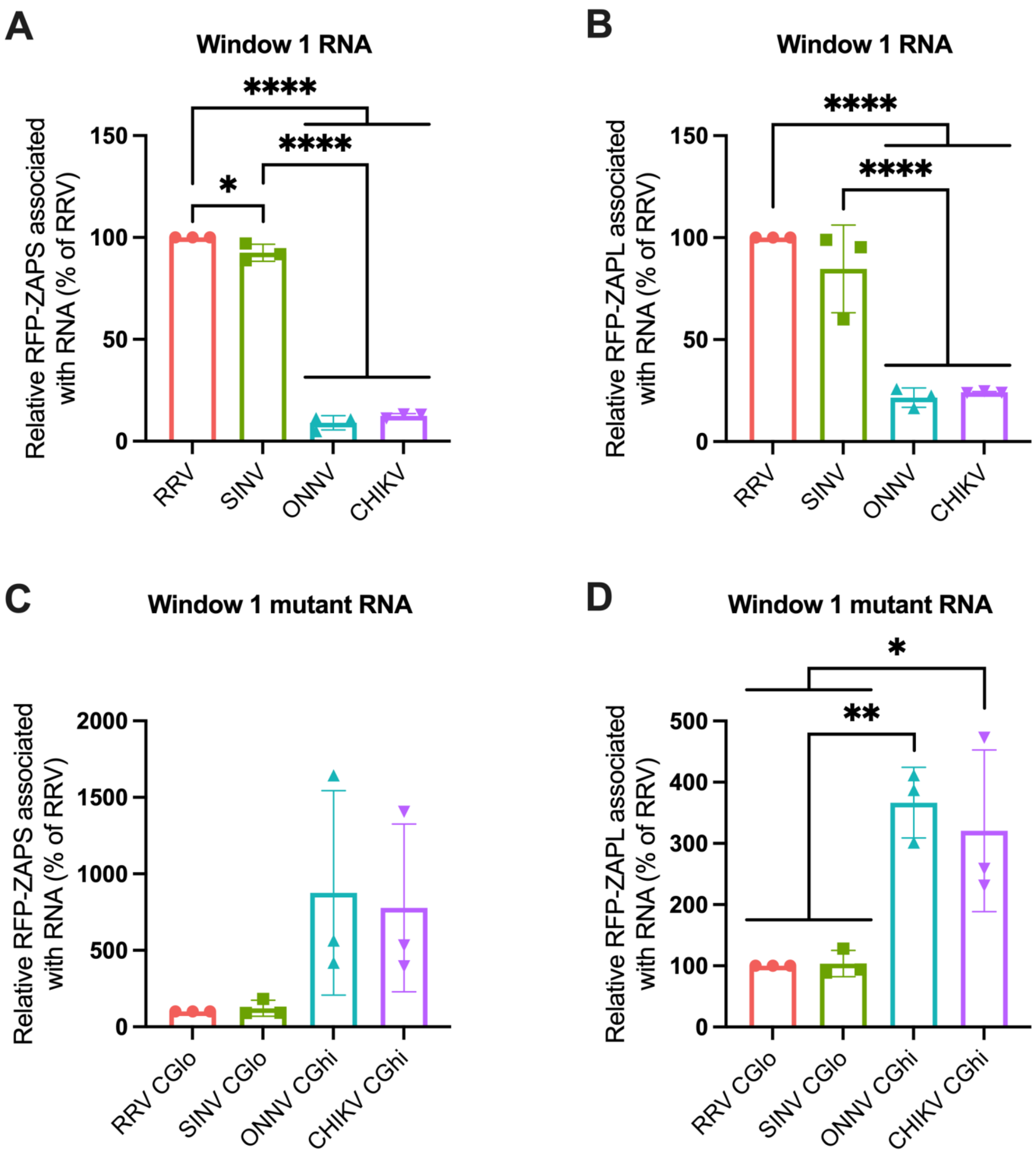

Figure S5. Quantifications of ZAPS and ZAPL binding to CpG-correlated window 1 RNA and CpG-correlated window 1 mutant RNA. ImageJ quantifications are shown of ZAP RNA pull-down assays for CpG-correlated window 1 RNA with RFP-ZAPS (**A**) or RFP-ZAPL (**B)** and for window 1 mutant RNA with RFP-ZAPS (**C**) or RFP-ZAPL (**D**). Data are combined from three independent experiments. Error bars represent the SD. Asterisks indicate statistically significant differences (one-way ANOVA and Tukey's multiple comparisons test: *, $p < 0.05$; **, $p < 0.01$; ****, $p < 0.0001$).

Supplementary Tables

Table S1. Primers for generation of EGFP-expressing CHIKV vaccine strain 181/clone 25.

Fragment	Origin	Sequence (5′-3′)	Forward (F) / Reverse (R)
Pre-GFP	CHIKV vaccine strain 181/clone 25	AGAGTGGCGGACCCGCTAAAAAGGTTATTTAAATTGGGCAAACCGTTAGCGG	F
		TCACCATGGCGCGCCTGATTGGTATTTAGGTACCTGCTG	R
EGFP	ONNV-GFP	TACCTAAATACCAATCAGGCGCGCCATGGTGAG	F
		CAAAGGTGGCCATGGACTAGTTTACTTGTACAGCTCGTCCATGCC	R
Post-GFP	CHIKV vaccine strain 181/clone 25	TACAAGTAAACTAGTCCATGGCCACCTTTGC	F
		GTTAAGGATGCTTGTAGCAGCTGATAGTACCCGGGGCTCATGACGTTGTCTT	R

Table S2. Primers to generate CpG-correlated window DNA templates for T7 transcription.

Virus	Window	Sequence (5′-3′, T7 promoter sequence in lowercase)	Forward (F) / Reverse (R)
RRV	window 1	taatacgactcactataggGGGCGATTTCAGCGCTAC	F
		GTCGCCTGTTTGATGGTAATATAT	R
	window 2	taatacgactcactataggTATTACGGCGTTGACTTAGAC	F
		CTGTACTCAGTGTGTATGTTC	R
	window 3	taatacgactcactataggGGCCAACGGTATGAATGTC	F
		GTCCTGAAGTTTCGGCCAC	R
	window 1 CGlo	taatacgactcactataggGGGAGGTTTCAGGGCTAC	F
		GTGGCCTGTTTGATGGTA	R
SINV	window 1	taatacgactcactataggCATACCTAAAGGAAACTTTCAGGC	F
		CTGTGCAGAGATAACTCTGGTTC	R
	window 2	taatacgactcactataggTTTGCATTAAGTTTTTCGGCATGGAC	F
		CACCAGGTCGTACCGTGCC	R
	window 3	taatacgactcactataggGGAGACAGGCACCGCAAG	F
		CTCCGCCATGTCTTTTGC	R
	window 1 CGlo	Same primers as for SINV window 1	
ONNV	window 1	taatacgactcactataggCAAAAGGGAACTTTAAGGCAAC	F
		GTTGGCCGGTATTATGTTGG	R
	window 2	taatacgactcactataggTACCCGCATATATGGGGTAG	F
		GAGTATGGATATTGATAACTACCAGG	R
	window 3	taatacgactcactataggCAATCATGTGCGGTCAATACCC	F
		GTTTGTTTTGGCCACATGG	R
	window 1 CGhi	taatacgactcactataggCAAAAGGGAACTTTAACGC	F
		GTTGGCGGGTATTATGTTG	R
CHIKV	window 1	taatacgactcactataggGGAAACTTCAAAGCAACT	F
		CCTGTTGACCGGTATAATG	R
	window 2	taatacgactcactataggGCGCATGTATGGGGTGGATC	F
		AAGGTGTGTGGATGTTTATGACCACTAG	R
	window 3	taatacgactcactataggGTGCGGTACGTATCCAGTAATCC	F
		CTCTGTTTGCTTTGGCCACATAG	R
	window 1 CGhi	Same forward primer as for CHIKV window 1	F
		CCTGTTGACGGGTATAATG	R

Table S3. qPCR primers.

Target	Sequence (5′-3′)	Forward (F) / Reverse (R)
ISG15	GGCTGGGAGCTGACGGTGAAG	F
	GCTCCGCCCGCCAGGCTCTGT	R
RPS11	GCCGAGACTATCTGCACTAC	F
	ATGTCCAGCCTCAGAACTTC	R

Table S4. CpG and UpA dinucleotide contents of CpG-correlated windows.

Virus	Window	CpG content (observed/expected)	UpA content (observed/expected)
RRV	window 1	0.970	0.838
	window 2	0.859	0.964
	window 3	0.906	0.967
	window 1 CGlo	0.357	0.880
SINV	window 1	0.864	0.867
	window 2	0.893	0.727
	window 3	0.828	0.744
	window 1 CGlo	0.446	0.838
ONNV	window 1	0.455	0.882
	window 2	0.523	1.092
	window 3	0.469	0.881
	window 1 CGhi	1.285	0.882
CHIKV	window 1	0.626	0.899
	window 2	0.639	1.269
	window 3	0.684	1.047
	window 1 CGhi	1.437	0.891
